# Towards a Graphene-Based Low Intensity Photon Counting Photodetector

**DOI:** 10.3390/s16091351

**Published:** 2016-08-23

**Authors:** Jamie O. D. Williams, Jack A. Alexander-Webber, Jon S. Lapington, Mervyn Roy, Ian B. Hutchinson, Abhay A. Sagade, Marie-Blandine Martin, Philipp Braeuninger-Weimer, Andrea Cabrero-Vilatela, Ruizhi Wang, Andrea De Luca, Florin Udrea, Stephan Hofmann

**Affiliations:** 1 Department of Physics and Astronomy, University of Leicester, University Road, Leicester LE1 7RH, UK; jsl12@le.ac.uk (J.S.L.); mr6@le.ac.uk (M.R.); ibh1@le.ac.uk (I.B.H.); 2 Department of Engineering, University of Cambridge, 9 JJ Thomson Avenue, Cambridge CB3 0FA, UK; jaa59@cam.ac.uk (J.A.A.-W.); aas73@cam.ac.uk (A.A.S.); mbcbm2@cam.ac.uk (M.-B.M.); pab96@cam.ac.uk (P.B.-W.); ac769@cam.ac.uk (A.C.-V.); rw520@cam.ac.uk (R.W.); ad597@cam.ac.uk (A.D.L.); fu10000@hermes.cam.ac.uk (F.U.); sh315@cam.ac.uk (S.H.)

**Keywords:** graphene, single photon, photodetector, visible, terahertz, cryogenic, X-ray

## Abstract

Graphene is a highly promising material in the development of new photodetector technologies, in particular due its tunable optoelectronic properties, high mobilities and fast relaxation times coupled to its atomic thinness and other unique electrical, thermal and mechanical properties. Optoelectronic applications and graphene-based photodetector technology are still in their infancy, but with a range of device integration and manufacturing approaches emerging this field is progressing quickly. In this review we explore the potential of graphene in the context of existing single photon counting technologies by comparing their performance to simulations of graphene-based single photon counting and low photon intensity photodetection technologies operating in the visible, terahertz and X-ray energy regimes. We highlight the theoretical predictions and current graphene manufacturing processes for these detectors. We show initial experimental implementations and discuss the key challenges and next steps in the development of these technologies.

## 1. Introduction

Single photon counting photodetectors require an incident single photon to be absorbed and to give a measurable signal. A number of different photodetector technologies have been developed for optical single photon counting with a wide range of specifications such as energy and time resolution, and operating temperature. For instance photomultipliers, avalanche diodes [1] and transition edge sensors [2] are able to operate with single photon resolution but without wavelength specificity in the optical range. Other detector technologies do exist that allow for single photon counting with optical wavelength specificity [3], but mostly operate at extreme cryogenic temperatures [4].

These detectors have many different applications, in areas as diverse as medical and space sciences or security applications. For instance a photon counting photodetector has applications on a satellite for the detection of faint, distant stars, or in fluorescence spectroscopy for use in characterizing biological samples. Single photon counting photodetectors also have quantum information applications, ranging from quantum key distribution (QKD) [5,6] to time-correlated fluorescence spectroscopy of quantum wells [7]. These new quantum applications are making significant demands on existing technologies due to the required signal to noise ratio, detection efficiency, spectral range and photon number resolution [8,9] .

Graphene is an allotrope of carbon, specifically arranged in a 2D hexagonal lattice structure with sp^2^ bonded carbon atoms. It has captured the world’s attention since it was first isolated in 2004 [10,11] due to a unique combination of mechanical and optoelectronic properties [11,12,13,14,15,16]. Graphene provides an interesting solution for single photon counting photodetection [17] with many potential applications; graphene has already been used for ultrafast photodetection on a femtosecond timescale [18] for pulsed lasers, its high carrier mobility enabling greater operational bandwidth. In addition, the tuneable band gap in bilayer graphene may enable sensitive photon counting photodetectors to operate with a trade off between resolution and operational temperatures, with resulting operational benefits.

## 2. Existing Technologies

A number of different techniques are currently utilised for single photon counting photodetection over a wide range of photon energies. For instance, a microwave kinetic inductance detector (MKID) passes a microwave through a circuit with a given frequency resulting in an inductance impedance through the circuit related to the frequency. A photon incident on a superconducting film (typically TiN) breaks Cooper pairs, creating additional charge carriers and changing the resonant frequency within the range 1–10 GHz [19]. To observe the change in phase and amplitude, very sensitive measurements are made before charge carriers recombine in time periods of order, 10^−3^–10^−6^ s. This technique has been used in detectors built into a 1000 pixel array [20]. MKIDs operate at temperatures ~100 mK [21] and have demonstrated position sensitivity with a noise equivalent power (NEP) of ~10^−17^ W·Hz^−1/2^ [22,23,24,25]. Ongoing research activities are being performed to investigate the use of graphene as an MKID [26,27].

Like the MKID, a superconducting tunnelling junction (STJ) can also be used for single photon counting at cryogenic temperatures. An STJ works by the absorbed photon energy breaking Cooper Pairs in a superconducting film, typically tantalum [22]. STJs have an effective band gap of order 1 meV, and operate at a low temperature, typically 300 mK, to ensure low dark noise. They have a time resolution of order microseconds and a typical resolution of order 1 eV for soft X-ray photons, and 0.1–0.2 eV for near-infrared and visible photons, with the Fano limit as the inherent energy resolution [22,23].

A number of different techniques have been proposed to allow low intensity photodetection at terahertz photon frequencies. Terahertz photodetection has been demonstrated using techniques such as bolometry [28], but many of these are at sub-THz frequencies. A technique using Photon Counting Terahertz Interferometry (PCTI) utilises the pulsed nature of photons at sub-far infrared frequencies, whereby detection on two or more telescopes can be used to measure the intensity correlation, enabling a wide bandwidth [29,30,31,32]. This technique requires detectors with a high count rate of 1–100 MHz and a time resolution better than 1 ps [31].

Table 1 provides a summary of the existing state of the art photodetectors for low intensity photon source illumination and for photon counting. Existing techniques, such as STJs and MKIDs, are able to count single photons, but have a timing resolution that is limited to approximately 1 µs. At similar photon wavelengths covered by the STJ, other detectors such as Avalanche Photodiodes and Transition Edge Sensors provide solutions. The Avalanche Photodiodes provide improved timing resolution but with compromised energy resolution. Transistion Edge Sensors provide less time resolution but improved energy resolution and very good responsivity. Across a wide range of wavelengths, microchannel plate photomultipler tubes provide an alternative to an STJ, with improved timing resolution up to ~25 ps, but with no energy resolution at optical wavelengths, and only very poor energy resolution at soft X-ray wavelengths. No detector exists that has the required combination of features for the current application demands of single photon counting photodetectors, such as high detection efficiency with wavelength specificity, high temporal resolution and low dark count [33].

Graphene-based photodetector techniques have been an exciting topic of research in recent years, with many potential applications in a number of different areas. The main detector techniques investigated are the photovoltaic effect, photo-thermoelectric effect, bolometric effect and the Dyakanov-Shur effect [24]. The photovoltaic effect exploits the separation of electron-hole pairs, with a resulting generation of a photocurrent between p and n doped areas. For the photo-thermoelectric effect, a photon absorption excites an e-h pair that leads to the ultrafast heating of the lattice, as this relaxes it induces a measurable photovoltage [56]. The increased temperature of the lattice can also be used for detection through bolometry due to a change in carrier conductance. The change in temperature is measured, with the thermal resistance also related to the power of the incident radiation [57]. Terahertz detection also exploits the Dyakanov-Shur effect, whereby radiation couples to the antennae, and excites a plasmon resonance between the contacts that generates a measurable DC photocurrent.

Field effect transistor detectors have been developed to exploit these detection mechanisms; for instance, graphene-based terahertz detectors have been developed by a number of groups [51,52,58], utilising many different photodetection techniques which usually require the coupling of the terahertz photon to the detector resulting in heating of the lattice or a plasmon resonance leading to a measurable photocurrent. These detectors have demonstrated excellent noise equivalent power (NEP) in the 10^−10^–10^−11^ W·Hz^−1/2^ range [52]. In addition, the Jovanovic group showed the development of a graphene field effect transistor (GFET) sensitive to X-ray photons, with silicon and silicon carbide absorbers and an applied back gate voltage [43,44,45,46,47,48]. These often require the photon to be absorbed in an absorber exciting multiple charge carriers that modulate the field applied to the graphene and resulting in a measurable change in the resistance. The Jovanovic group found that it was not possible to obtain an X-ray signal at room temperature for highly resistive silicon, only at 4.3 K [44]. Additionally it can be shown that a significant energy is required for a measurable change in resistance, with a signal rise time of order of seconds, which makes this technique currently not suitable for a single photon counting photodetector. However the change of measured resistance of a graphene field effect transistor-like structure has already been shown to enable sensitive detection of single molecules [59] suggesting that single photon sensitivity is feasible. In addition, work by Xia et al. [50] has shown sensitivity to 1.55 µm laser illumination with a 3 mW energy deposition, leading to an experimentally determined bandwidth of 40 GHz, compared to the theoretically predicted maximum of 500 GHz. Other novel field effect detectors have potential, such as a black phosphorus-zinc oxide nanomaterial heterojunction with a reported on/off ratio of 10^4^ and no time delay [54].

Detectors with wavelength specificity such as the MKID and STJ detectors require cryogenic cooling to prevent dark noise that is critically dependent on the energy gap in the Cooper pairs for both techniques. Varying this energy gap by means of graphene’s tuneable band-gap would enable potential operation at higher temperatures, overcoming cost and operational issues of cryogenic cooling. Scope also exists to exploit graphene to develop further high speed photodetectors for different photon energies with possibility for femtosecond photodetection [18], and to enable PCTI with smaller pixel sizes to allow for greater resolution resulting from a greater pixel density [29,30,31,32].

Table 1 highlights the already impressive characteristics of graphene-based photodetectors using a number of different techniques, suggesting that it may provide a potentially interesting and viable solution to future technologies. Throughout the rest of this paper we will outline how graphene can be applied to such future single photon counting technologies, with a particular focus on the devices that we are developing. In Section 3 we outline the critical properties of single and bi-layer graphene for photodetection. In Section 4 we consider our theoretical study of bilayer graphene as a single photon counting photodetector at visible wavelengths, and in Section 5 we discuss our studies working towards a detector optimised for operation at a frequency of 1.2 THz. In Section 6 we discuss our progress to develop an X-ray detector at room temperature and suggest potential iterations to the design. This motivates our discussion in Section 7, where we consider the latest state of the art for graphene device fabrication, its limitations, and possible future solutions.

## 3. Properties of Single and Bilayer Graphene

**Figure 1:**
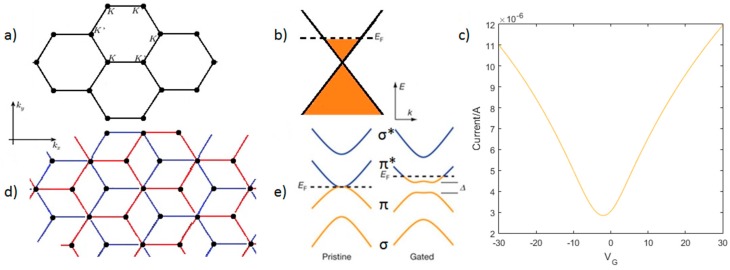
Showing (**a**) the honeycomb structure of single layer graphene with the K and K’ points in the first Brillouin Zone (reproduced by permission of Cambridge University Press, subject to cambridge.org/uk/information/rights/permission.htm); (**b**) the linear energy-wavenumber relationship close to the Dirac point with a Fermi level that we can change through the application of a electric field; (**c**) the drain-source current of graphene against gate voltage [74] with a sample dependent Dirac point and electron and hole mobilities; (**d**) the structure of AB stacked bilayer graphene, with the two layers marked in red and blue respectively and a hopping parameter of ~0.4 eV between the layers; and (**e**) the band structure for bilayer graphene showing pristine bilayer graphene and the opening of a band gap for gated bilayer graphene with AB stacking (reproduced with permission of Nature Publishing Group) [75]. In the gated we see “trigonality” at very low energies [16].

Graphene has many properties that make it promising to the development of new photodetector technologies and potentially outperform other existing materials. The low energy band structure of graphene is dictated by π states which form symmetrical cones touching at the so called Dirac point (Figure 1a). Graphene is therefore usually described as zero-bandgap semiconductor. The electron dispersion in this region is linear (Figure 1b), reminiscent to that of light and unlike conventional parabolic dispersions in semiconductors. The band structure is symmetric about the Dirac point, i.e., electrons and holes should have the same properties. The Fermi velocity is calculated to be approximately 10^6^ ms^−1^ [12,16,60]. Graphene can support very high carrier mobilities (10^6^ cm^2^·V^−1^·s^−1^ for suspended graphene at temperatures ~5 K [13] to higher temperatures [14]) but, as with most of its properties, this strongly depends on the environment and support. Fully encapsulated graphene devices on silicon/silicon dioxide support show mobilities in the order of 10^3^ cm^2^·V^−1^·s^−1^ at room temperature [61]. High carrier mobilities offer the potential for an ultrafast detector; photodetection has been demonstrated at femtosecond resolution [62], with GFETs developed with a theoretical bandwidth up to 500 GHz [50].

The carrier density (or doping level) of graphene is continously tunable from p-type to n-type through charge transfer, often unintentionally due to external factors such as air exposure and substrate effects. Due to this high sensitivity, reproducibility of electrical characteristics is a key challenge which may be addressed by considering techniques such as encapsulation [61,62,63] to reduce atmospheric effects or controlled doping [64,65]. We can also exploit the change of doping through the field effect, whereby a field applied to the graphene shifts its Fermi level [43] and hence changes the number of charge carriers and therefore the conductivity of the graphene [47,48,66,67,68,69]. In Figure 1c we see the change in conductivity resulting from the application of a gate voltage for four different samples, with hole transport and electron transport at negative and positive gate voltages respectively. At gate voltages far from the Dirac point we obtain a linear conductivity-gate voltage relationship, with the gradient related to the carrier mobility of the sample [70]. Employing the field effect has enabled detection of X-rays with a relatively simple device fabrication and detector measurements [43,44,45,46,47,48].

Graphene has a wideband absorption of 2.3% [15] per layer at visible frequencies, although this leads to low photoresponsivity and low external quantum efficiency (EQE) [71,72,73]. However it is possible to exploit plasmonic nanostructures to improve this EQE, a technique that has been shown to enhance the photocurrent by up to 1500% [73]. Interestingly, we can exploit the production of plasmons to enable terahertz photodetection by utilising the Dyakanov-Shur effect [51,52]. In this technique terahertz radiation is coupled into an antennae resulting in the excitation of plasmon waves in a graphene channel and the generation of a measurable DC photocurrent.

Flexible graphene-based photodetectors using centimetre-scale grown samples have also been developed. In [76] the authors report an internal responsivity of 45.5 AW^−1^ and internal responsivity of 570 AW^−1^ for a laser source intensity of 0.1 nW·µm^−2^ and maintain this photodetection down to a bending radius of 6 cm.

Bilayer graphene is also of interest in the development of photodetector technologies. For bilayer graphene the crucial additional parameter is the stacking of the two layers [75]. For instance AA stacked graphene has the two layers directly above each other, whereas AB (Bernal) stacking has an offset in the arrangement as shown in Figure 1d. The layer interactions change the band structure, as highlighted in Figure 1e for AB-stacking, which shows a hyperbolic (non-linear) bandstructure. An approach for opening a tunable band gap for such bilayer graphene is to apply an electric field perpendicular to the layers (Figure 1e) [75], a technique that shows no hysteresis and also allows tuning of the Fermi level. The band gap magnitude is given by $U_{g}=\frac{\left|U\right|\gamma_{1}}{\sqrt{\gamma_{1}^{2}+U^{2}}}$, where $U_{g}$ is the band gap, U is the interlayer asymmetry and $\gamma_{1}$ is the interlayer hopping parameter; the magnitude of the band gap saturates $U_{g}\to \gamma_{1}$ for large U [77]. Other techniques that have been reported to open a band gap include the controlled adsorption of water [78] or hydrogen [79], applying strain [80], and molecular doping [81].

## 4. Bilayer Graphene Single Photon Counting Photodetector—Simulations and Design

Our work considers the application of a potential, V, applied perpendicularly to the lattice [16,75]. This breaks the interlayer symmetry and leads to the electron energy spectrum [16] given by:

(1) $$E^{2}=\gamma_{0}^{2}{\left|S\left(k\right)\right|}^{2}+\frac{\gamma_{1}^{2}}{2}+{\left(\frac{V}{2}\right)}^{2}\pm \sqrt{{\left(\frac{\gamma_{1}^{2}}{2}\right)}^{2}+\left(\gamma_{1}^{2}+V^{2}\right)\gamma_{0}^{2}{\left|S\left(k\right)\right|}^{2}}$$

as described in Figure 1e [75], where $\gamma_{0}$ = 2.97 eV and $\gamma_{1}$ = 0.4 eV [16] are the intralayer and interlayer hopping parameters respectively and:

(2) $$S\left(k\right)=\underset{\delta }{\sum }e^{\mathrm{ik}\delta }=2\exp\left(\frac{{\text{ik}}_{x}a}{2}\right)\cos\left(\frac{k_{y}a\sqrt{3}}{2}\right)+\exp\left(-{\text{ik}}_{x}a\right)$$

where k is the wavevector and a=1.42 Å is the near neighbour distance [16].

As bilayer graphene possesses a variable band gap [75], unlike many other materials including single layer graphene, it allows the potential for a detector that can exploit this tuneability to vary the resolution for optimal performance.

Initially, we developed a number of simulations for our bilayer graphene single photon counting photodetector, which indicate the fundamental operational properties and parameters of the detector. We firstly calculate the density of states and investigate the optimum operational window [82]. We then use a Monte Carlo simulation using a Gillespie Algorithm [83] to simulate the absorption of an incident photon on the graphene lattice, the excitation of a photoelectron and its subsequent relaxation in the conduction band.

### 4.1. Density of States and Optimum Operational Window

**Figure 2:**
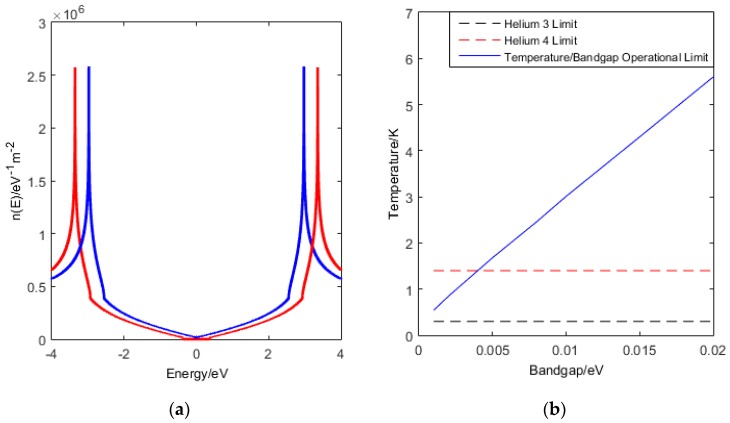
Showing (**a**) the density of states for bilayer graphene with a band gap of 5 meV; red is the σ band, blue is the π band. (**b**) shows the o perational limit of a bilayer graphene photodetector. In this simulation, A = 1 mm^2^. Helium-4 cooling limit is 1.4 K and Helium-3 limit is 0.3 K.

Firstly we calculate the density of states, n(E), numerically (Figure 2a) and integrate the Fermi-Dirac distribution over the first Brillouin zone to determine the number of charge carriers in the conduction band per unit area given by:

(3) N=∫0Ephoton2dE1exp(EkbT)+1n(E)

where E is the electron energy and T is the temperature. The integration limit given by Ephoton2 arises from the possible photon excitations from the valence band to the conduction band at energies we are interested in.

For a single photon counting photodetector we require it to be statistically unlikely that electrons are thermally excited into the conduction band. We therefore calculate numerically NA, where A is the sample area, and look for cases where NA=1, as plotted in Figure 2b. Below this line, NA<1, is the regime where there is theoretically no dark current. This is critically dependent on the bilayer graphene density of states. The tuneable band gap in bilayer graphene allows us to exploit this operational limit, as this approach allows us to run our device at higher temperatures, with a larger band gap, but with a trade off against energy resolution.

### 4.2. Monte Carlo Simulations

We have developed a Monte Carlo simulation to determine the likely properties of our photodetector [82]. Our model assumes that we operate within the limit shown in Figure 2b, i.e., electrons in the conduction band result solely from the initial photoexcitation (or subsequent relaxations). Furthermore, excitation occurs when the photon energy is equal to the energy difference between two bands in the valence and conduction bands respectively shown in Figure 1e.

After the initial excitation, the electron can relax through a number of different relaxation paths. For instance electron-electron scattering (EES) is the inelastic scattering between two electrons in the conduction band (CB) and does not affect the total energy or the number of electrons in the CB. Another possibility is electron-phonon scattering (EPS) which is the scattering of an electron due to the emission (absorption) of a phonon to (from) the lattice [84], resulting in energy lost (gained) from the electrons. Alternatively the electron may relax through impact ionisation (II) or Auger recombination (AR); II is the excitation of an electron from the valence band (VB) to the CB due to the loss of energy from a CB electron. In this model II is the only process which results in an increase in the number of electrons in the conduction band [85,86,87]. AR is the reverse process, where an electron relaxes from CB to VB, when another CB electron becomes more excited. At low temperatures, the rates of electron-phonon scattering, $\sigma_{\text{Phonon}},$ and electron-electron scattering, $\sigma_{E-E}$, are given respectively by [88]:

(4) $$\sigma_{\text{Phonon}}=\sigma_{\text{Acoustic}}+\sigma_{\text{Optical}}\;\approx \frac{D_{0}^{2}}{\rho_{m}\omega_{0}{\left(\hbar v_{F}\right)}^{2}}\left[\left(E_{k}-\hbar \omega_{0}\right)\left[\frac{1}{e^{\frac{\hbar \omega_{0}}{k_{B}T}}-1}+1\right]\theta \left(E_{k}-\hbar \omega_{0}\right)+\left(E_{k}+\hbar \omega_{0}\right)\left(\frac{1}{e^{\frac{\hbar \omega_{0}}{k_{B}T}}-1}\right)\right]$$

(5) $$\sigma_{E-E}=\frac{1}{\tau_{\text{MFT}}}=\frac{v_{F}}{\lambda }=2k_{f}\frac{\hbar k_{f}}{m_{e}}=\pi n\frac{2\hbar }{m_{e}}$$

where $\omega_{0}$ is the phonon frequency, $E_{k}$ is the electron energy, T is the temperature, $\rho_{m}$ is the mass density, D_0_ is the deformation potential constant, λ is the wavelength, $v_{F}$ is the Fermi velocity, $k_{f}$ is the Fermi wavenumber and n is the density of charge carriers.

**Figure 3:**
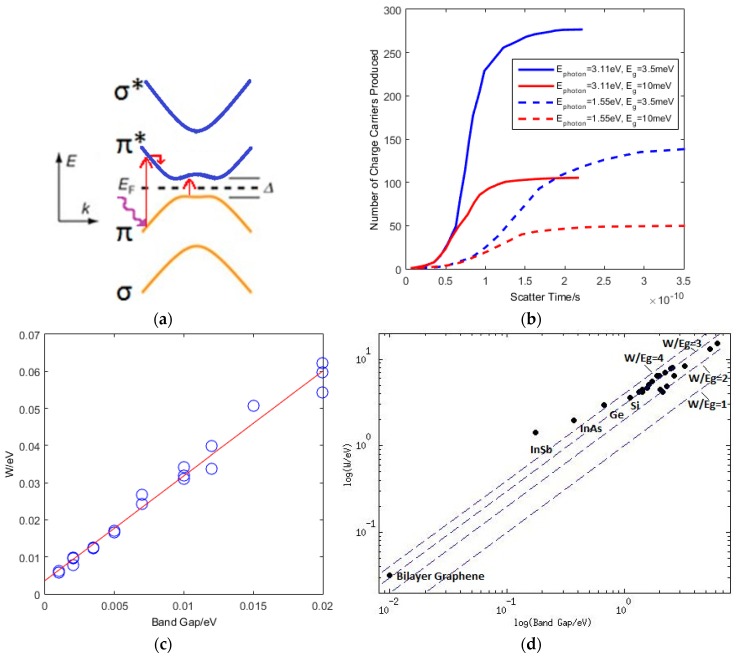
(**a**) Schematic to show the absorption of a photon and the excitation and subsequent relaxation the hot photoelectron; (**b**) The distribution N(t) at $E_{\text{gap}}$ = 1 meV and 3.5 meV for photons with energy 3.11 eV and 1.55 eV, and with μ = 100; (**c**) Electron-hole pair creation energy as a function of band gap with μ = 100. The circles show simulation results, and the red line is best fit straight line; (**d**) Comparison of W vs. band gap for bilayer graphene with other semiconductors.

In the literature, little work has been done on the analytical II and AR rates for low CB electron density at low temperature. However, as we start with only one conduction band electron following the photoexcitation, we assume that EES, EPS and AR relaxation rates will be significantly lower than II as the former are CB density dependent, whereas II is VB density dependent [86]. Furthermore, relaxation rates at lower energies such that electrons relax out of CB altogether are low, due to the necessity to conserve energy and momentum whilst filling vacant holes in the VB from previous electron excitations. Therefore in the low electron density, low temperature limit, II highly dominates. To run simulations we choose a ratio, µ, of phonon scattering rate to impact ionisation rate, where II dominates. We run simulations with each of the relaxation events chosen randomly, weighted based on the relevant rates, and solve numerically to find solutions where energy and momentum are conserved. We test the dependence of the number of charge carriers produced as a function of time, initial photon energy, band gap and μ=σIIσPhonon. In our simulations we use the interlayer hopping parameter $\gamma_{1}$ = 0.4 eV. A schematic of this is shown in Figure 3a.

We ran our first simulations over a given time, at different initial energies and different band gaps, as shown in Figure 3b. The results show, as anticipated, that the number of electrons produced increase with initial energy. Additionally, as the band gap is increased the number of electrons produced is significantly reduced.

By simulating with different size band gaps and photon energies we calculate the average electron-hole pair creation energy, $W=\frac{E_{\text{photon}}}{N}$, as shown in Figure 3b. This gives a W to band gap ratio of 3–4, similar to that of semiconductors such as silicon and germanium (Figure 3d) [89].

**Figure 4:**
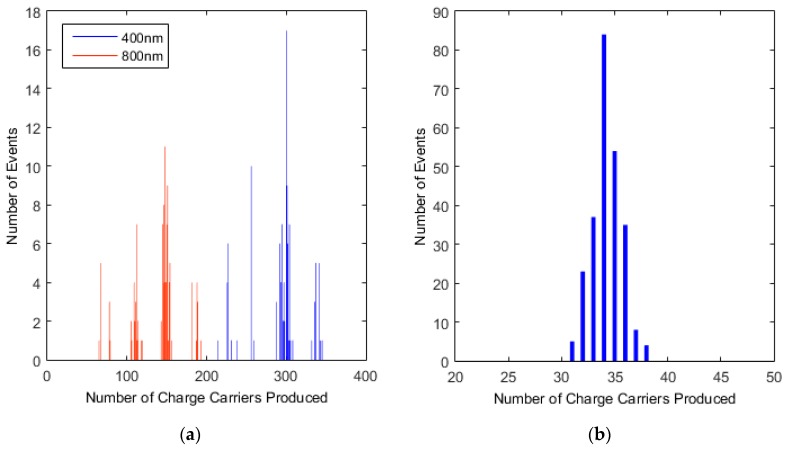
Showing (**a**) the number distribution as a function of photon energy for μ = 100; and (**b**) the distribution of events for λ = 3500 nm photon. μ = 100.

A plot of the dependence on the initial photon energy of the distribution of charge carriers produced is shown in Figure 4a. Clearly, for a more energetic photon, more electrons will be produced. We observe wavelength specificity as the difference in the distributions at each wavelength. Additionally, in Figure 4a we see four peaks in the simulations, caused by the four alternative excitations from the π and σ bands to the π* and σ* bands respectively. The gap between the centre of the peaks is equal to $\Delta N=\frac{\gamma_{1}}{W}$, where $\gamma_{1}$ is the hopping parameter between layers, and W is the average ionisation energy. The characteristic peak of an event is highly dependent on the initial transition between the bands, and the initial relaxation step. The presence of the four peaks makes energy resolution of the incident photon problematic.

However for a photon energy less than $\gamma_{1}$ = 0.4 eV (i.e., in the IR spectrum), we obtain only one peak since the lower available energy allows only one possible transition. Figure 4b shows, with λ = 3500 nm and a band gap of 3.5 meV, that we get one large peak in the distribution, with a W value still in the range, 3–4, as also seen at visible wavelengths.

**Figure 5:**
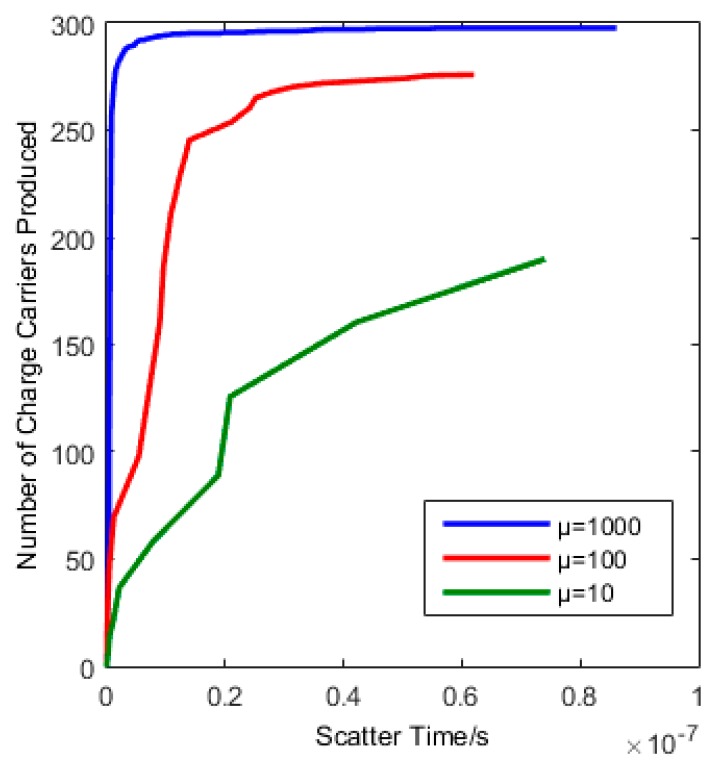
A plot indicating the number of charge carriers versus time showing the effect of changing the II rate.

Initially we arbitrarily picked the II rate by using a ratio to the phonon rate, μ. We then tested the effect of changing the ratio to ensure that the total number of charge carriers produced tends towards the same value, but at an increased time, with decreasing values of the ratio. The results are shown in Figure 5 for a photon with λ = 400 nm and a 3.5 meV band gap.

If we integrate over the entire active scattering time (i.e., the time during which electrons continue to relax and collect at the bottom of the conduction band) then this gives us an estimate of the total number of charge carriers produced. The II rate is then indicative of the active scattering time, with an active scattering time of order 10^−8^ s illustrated in Figure 5.

**Figure 6:**
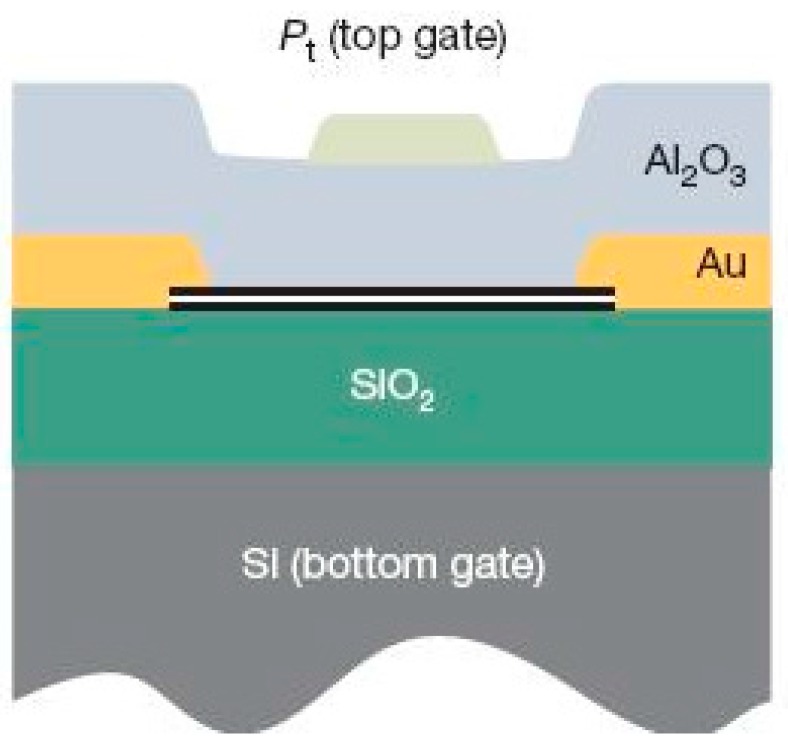
Schematic used in [75] that has been used to show opening of a tuneable bandgap in bilayer graphene. Our bilayer graphene detector design is based on this schematic (reproduced with permission of Nature Publishing Group).

Our results enabled us to design our prototype detector, based on the schematic from [75], Figure 6, where they first demonstrated a tuneable band gap in AB-stacked bilayer graphene. Our prototype single pixel detector design has a silicon substrate with a 300 nm thick silicon dioxide insulating layer. Ni-Al contacts are deposited on top of the graphene in order to provide electrical connections to the graphene, with a top gate dielectric of alumina deposited through atomic layer deposition (ALD). For the top gate, indium tin oxide (ITO) contacts are deposited; indium tin oxide is typically used in transparent electronics and is opaque at UV photon energies but is transparent at visible photon energies [90].

In summary, our results demonstrate the feasibility of a new type of ultrafast photon counter operating at optical and IR wavelengths. Such a device can be operated at approximately 100 MHz, although higher frequencies may be possible with improved calculations of the impact ionisation rate to give our detector comparable or superior results to other detectors. We obtain a value of the electron-hole pair creation energy, W, as a function of the band gap. The ratio between W and the band gap is found to be comparable to that of other detectors such as Si and Ge [89]. The detector has scope to enable a trade-off between operating temperature and energy resolution, allowing for a cryogenic single photon counting photodetector to operate at temperatures that do not require helium-3 cooling albeit with reduced energy resolution. This approach could enable a lower cost detector to be developed for space science where extreme levels of cooling are complex and expensive.

## 5. Dyakonov-Shur GFET Optimised for 1.2 THz—Simulations and Design

A number of different techniques can be used for photodetection at terahertz frequencies, such as the photothermoelectric effect and bolometry [62]. Another technique is the Dyakonov-Shur effect, whereby a terahertz photon impinges on GFET contacts, designed as antennae, and excites a plasma wave that resonates between the source and the drain of the channel that gives a non-linear photoresponse as a DC voltage [91]. We base our detector on this technique, and utilise simulations discussed in [51] to design our detector and optimise the parameters for the regime that we are interested in.

**Figure 7:**
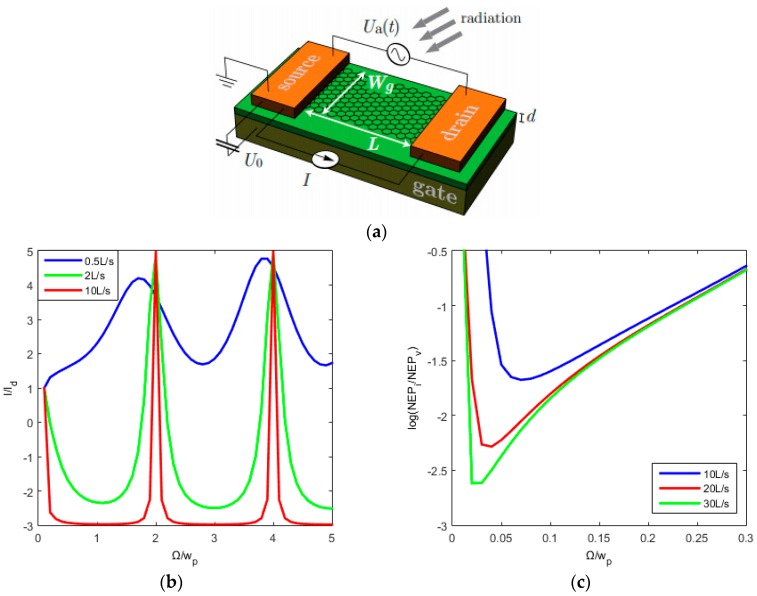
(**a**) The layout of a GFET utilising the Dyakonov Shur Instability (reproduced from [51] with permission of AIP Publishing under a Creative Commons license subject to https://publishing.aip.org/authors/rights-and-permissions); (**b**) the photocurrent against the photon frequency for different momentum relaxation time, where s is the plasma wave speed; and (**c**) $\log\left(\frac{{\text{NEP}}_{I}}{{\text{NEP}}_{V}}\right)$ against photon frequency with lower noise at higher momentum relaxation time.

Tomadin [51] discusses a THz detector in a FET structure, with an AC potential $U_{a}$ generated between the source and drain and the back gate, kept at a voltage $U_{0}$ relative to this. The graphene is of length L between the gates and width W, with a substrate thickness, d. This design, illustrated in Figure 7a, measures the generated photocurrent I which is related to the energy of the incident photon. The photocurrent generated between the contacts is given by:

(6) $$\frac{I}{I_{d}}=1+2\beta \left(\omega \tau \right)F\left(\omega ,\tau \right)$$

where $I_{d}$ is the diffusive current, $\beta \left(x\right)=\frac{2x}{\sqrt{1+x^{2}}}$, ω is the frequency of the incident photon, τ is the momentum relaxation time:

(7) $$F\left(\omega ,\tau \right)=\frac{\cos h\left(\left(2K_{2}L\right)\right)+\cos\left(2K_{1}L\right)-2}{\cos h\left(2K_{2}L\right)-\cos\left(2K_{1}L\right)}$$

and $K_{1}$ and $K_{2}$ are the real and imaginary parts of the wave number K respectively.

In Figure 7b,c, $\frac{I}{I_{d}}$ and $\log\left(\frac{{\text{NEP}}_{I}}{{\text{NEP}}_{V}}\right)$ are plotted against $\frac{\Omega }{\omega_{P}}=\frac{2L\Omega }{\pi s}$ respectively, where Ω is the frequency of the incident THz radiation, $\omega_{P}=\frac{\pi s}{2L}$ is the resonant plasma angular frequency, s is the plasma wave velocity, L is the length of the graphene channel, NEP is the noise equivalent power and $\frac{{\text{NEP}}_{I}}{{\text{NEP}}_{V}}$ is the ratio between the current noise and voltage noise [51]. Figure 7b shows that we see a peak in the I/I_d_ which becomes increasingly sharper with increasing momentum relaxation time, and at regular values of Ω/ω_p_. For larger momentum relaxation time we also see a lower noise, Figure 7c. These plots show that we can pick a number of solutions for the parameters of our detector designed for detection of photons with a frequency of 1.2 THz and potentially provide results which are measurable and realistic. In addition, as outlined in [51], by varying $U_{0}$ it is possible to control the Fermi level, plasma wave speed, fundamental plasma angular frequency and diffusive photocurrent. Therefore, by changing $U_{0}$, we can maximize the photocurrent for a given photon frequency, trade off the noise for optimised device response, and enable a degree of tuneability to maximize the response of the detector over the wide frequency range of interest.

**Figure 8:**
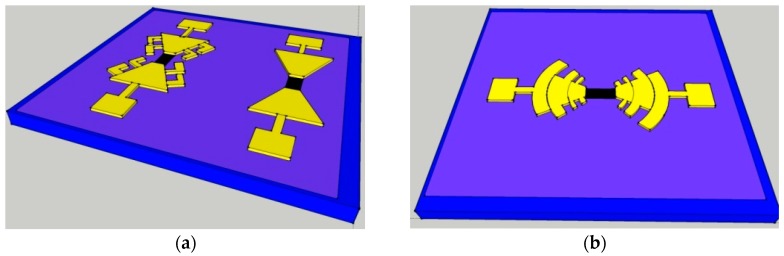
(**a**) The “beetle” (left) and bowtie (right) antennae and (**b**) the log-periodic, circular toothed antenna (not to scale). Both these were designed to have a resonating frequency at the required 1.2 THz. Here blue is Si, light purple is SiO_2_, yellow is the Ni-Al contact and black is the graphene.

Across our devices the graphene channels were coupled to a number of different antennae, either a bowtie (or a variant “beetle” antenna) as shown in Figure 8a, or a log periodic circular toothed antenna shown in Figure 8b. These were optimised using Sonnet Lite simulation software to resonate at the required frequency range. The schematics show a silicon back gate with a 300 nm thick silicon dioxide insulator between the silicon and a graphene channel of 10 µm × 5 µm, with the graphene channel etched to the required dimensions and nickel-aluminium deposited for the contacts and antennae. The antennae are of order 100 µm from the graphene to the edge of the antennae, with the ratio between the arms of the electrode set to 1.5. This means we operate in the long gate regime, discussed further in [92], where plasma waves have been shown to be excited.

## 6. X-ray Graphene Field Effect Transistor

**Figure 9:**
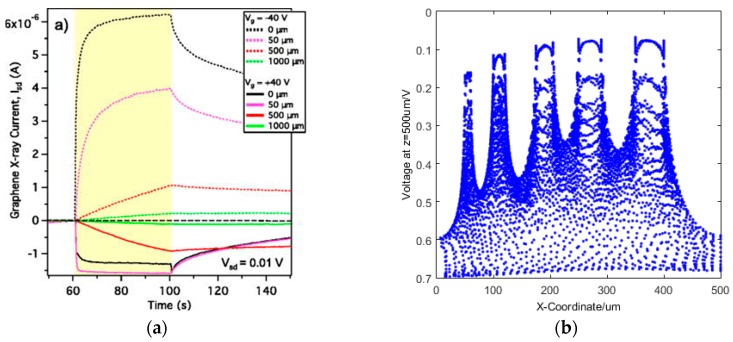

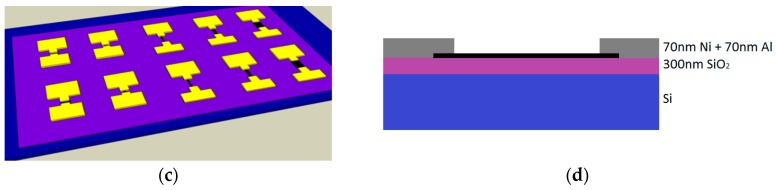
(**a**) The signal with illumination by a 10.1 µW X-ray source over time for a graphene FET based on a silicon carbide substrate (reproduced from [49] with permission of AIP Publishing under a Creative Commons license subject to https://publishing.aip.org/authors/rights-and-permissions). This shows slow illumination and slow decay for an X-ray photon beam, The same group have also shown the sensitivity of a graphene FET based on a silicon substrate at 4.3 K [48]; (**b**) the funnelling of charge carriers towards the substrate dielectric with the application of a gate voltage. In this simulation the charge carriers are funnelled towards 5 contacts on the substrate surface of increasing size; (**c**) shows the the design of an X-ray GFET test device; here blue is Si, light purple is SiO_2_, yellow is the Ni-Al contact and black is the graphene of different channel sizes for each device, with another, side-on, schematic shown in Figure 9d.

A number of groups are working on the development of graphene-based X-ray detectors using a number of different techniques. The most promising developments are from the Jovanovic group [43,44,45,46,47,48,49], where they have showed a graphene field effect transistor on a silicon carbide structure at room temperature. They have also demonstrated sensitivity to an X-ray photon beam (15 kV, 15 µA and 40 kV, 80 µA) for an undoped silicon substrate, but only at 4.3 K [48]. This has shown good energy resolution, of order $\frac{E}{\delta E}\sim 10,000$ with contributions from the number of charge carriers produced and limitations due to device design [43]. They have also shown a responsivity of 0.1 AW^−1^ but has presented difficulties with regards to the speed of detection. As shown in Figure 9a the illumination time is ~40 s, with a signal decay time of seconds for both the silicon carbide and silicon respectively. This technique works by modulating the charge carrier density in the substrate, with a resulting change in the resistance of the graphene.

The detectors developed by the Jovanovic group would be unsuitable for high speed, low intensity single photon counting photodetection, but the graphene channel resistance technique, which has also been shown capable of single molecule sensing [59], potentially provides a good basis for our X-ray single photon counting photodetector. For our prototype detectors we have used a silicon substrate with a conductivity of ~100 Ω·cm and operating at room temperature. An incident X-ray photon is absorbed by the silicon, with the resulting electron-hole pair scattering through the silicon directed by the application of a field to funnel the charge carriers to the substrate dielectric as shown in Figure 9b. This build up of charge develops a field across the substrate and applies a field to the graphene resulting in a change in the channel resistance. Our test chip consists of CVD grown graphene that was transferred onto a silicon substrate with resistivity ~100 Ω·cm and a 300 nm thick silicon dioxide insulating layer.

The graphene channels were etched to different sizes from 5 µm × 10 µm up to ~50 µm × 100 µm. These were connected to nickel-aluminium source and drain pads, as shown in Figure 9c,d; nickel obtaining low contact resistance with the graphene and aluminium for better wirebonding.

**Figure 10:**
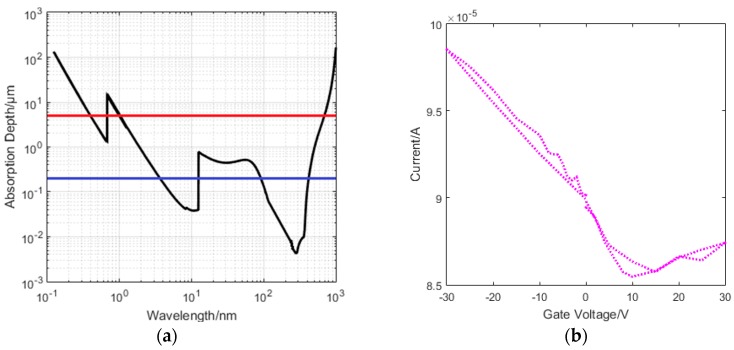
(**a**) the wavelength dependence of photon absorption length for silicon. The red and blue lines show the absorption depth at 650 nm and 405 nm laser wavelengths respectively; and (**b**) showing the current as a function of the gate voltage with the Dirac point at approximately 10 V.

Whilst our eventual aim is to detect low intensity or single photon sources, we chose to undertake initial experiments using illumination from a pulsed optical laser to characterise the behaviour of the detector and, in particular, its likely sensitivity. These pulsed lasers were calibrated using an Excelitas C30742-33 Series silicon photomultiplier (SiPM). The pulsed laser offers many advantages for initial characterisation including simple control of the deposited energy via variation of pulse width or by attenuation with filters, as well as providing a periodic strobe signal with which the detector output pulse, if present, will be synchronised cf. the unknown random arrival time of X-ray events from an X-ray source. The latter capability is critical when trying to measure the sensitivity while looking for the smallest detectable pulse above the noise. In addition the laser pulse can be used to generate a deposited energy at equivalent depths in the substrate to UV and soft X-ray single photons. Figure 10a shows the wavelength dependence of the photon absorption depth in silicon, the red and blue horizontal lines indicating the absorption depths at 650 nm and 405 nm respectively, showing that the red laser absorption depth is analogous to soft X-rays ~1–4 keV. The device was characterised by applying a 10 mV source-drain voltage, and varying the back gate voltage whilst measuring the source-drain current. The device has a Dirac point at approximately 10 V gate voltage, as shown in Figure 10b.

**Figure 11:**
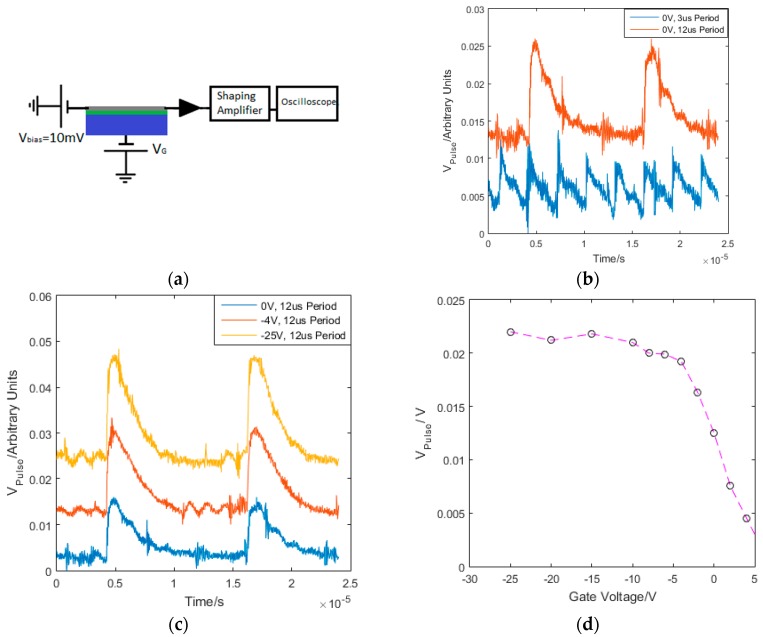
(**a**) the current sensitive preamplifier arrangement with the graphene (grey), SiO_2_ (green) and Si (blue), with a 10 mV bias between the source and drain of the graphene and the output from the current sensitive preamplifier output on the oscilloscope (**b**) the dependence of the detector signal on the laser pulse frequency and (**c**) the gate voltage applied. N.B. periodic noise at ~200 kHz is also apparent. The detector signal has a very fast rise time and a fall time linked to the recombination time of the charge carriers in the silicon. (**d**) shows the dependence of the signal pulse height on the gate voltage, with a saturation point at approximately −10 V attributed to limits on carrier transport in the Si given by SRH recombination.

The device was initially connected to an Analog Devices ASA4817-1 amplifier in transimpedance mode, with V_bias_ = 10 mV voltage applied between the source and drain contacts, as shown in Figure 11a. The device was then illuminated by a pulsed optical laser with a wavelength of 650 nm and pulse width down to 40 ps. Following the illumination of the detector, the current sensitive preamplifier detects the change in source-drain current and provides a voltage output, V_Pulse_, captured on an oscilloscope. Figure 11b,c show the dependence of the detector signal on pulse frequency and back gate voltage respectively. Figure 11d shows that the peak amplitude increases for increasing negative gate voltages, until saturating at approximately −15 V.

**Figure 12:**
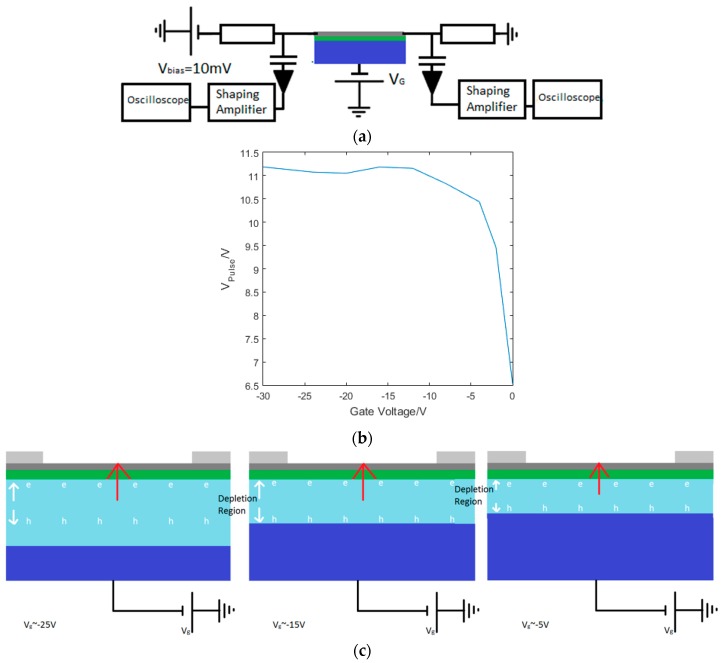
(**a**) shows the charge sensitive preamplifier arrangement with a resistor each side of the graphene to give a voltage change that gives a measurable change in charge due to the presence of the capacitor; and (**b**) show the dependence on the gate voltage in the charge sensitive preamplifier arrangement, with saturation in the pulse amplitude that we again attributed to limits on carrier transport in the silicon given by SRH recombination. (**c**) shows a schematic for the detection mechanism, with the dipole between the electron and hole pair larger for larger depletion regions until they become limited by the SRH recombination time. The dipole created causes a field that changes the graphene conductivity.

In order to reduce the noise for higher sensitivity we rearranged the GFET measurement circuit. The device was connected to two low noise, high gain, Canberra 2001 charge sensitive preamplifiers on each contact coupled through two 1 pF capacitors, with two 1 kΩ resistors in series with the graphene, as shown in Figure 12a. Conceptually, when the detector is illuminated, the resistance of the graphene and therefore the voltage across the graphene varies creating a voltage pulse between graphene and the resistor, which in the presence of the capacitor creates a charge pulse which is measured by the the charge sensitive preamplifier, and outputs a voltage pulse, V_Pulse_. In this arrangement we identified the same saturation as in Figure 11d, which is shown in Figure 12b.

**Figure 13:**
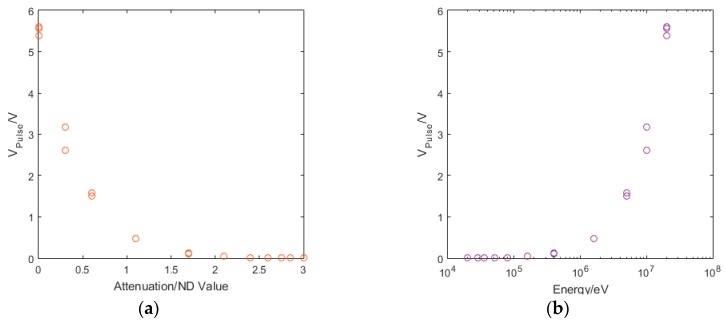

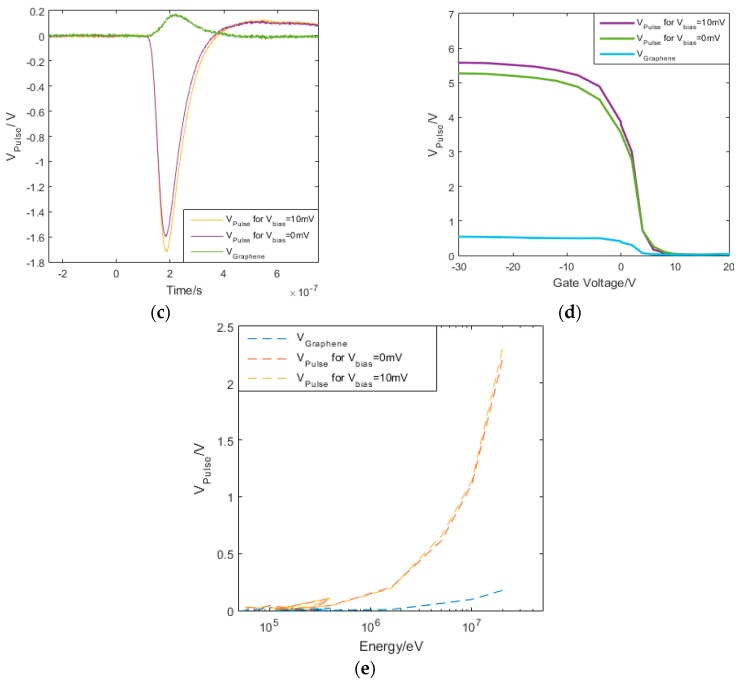
Showing (**a**) the exponential decay on the signal with increasing attenuation of the laser input; (**b**) the measured pulse energy collected in the absorber, indicating an energy sensitivity to ~100 keV; (**c**) the difference in the pulse with Vbias = 10 mV and 0 mV for a gate voltage of −15 V showing a small peak attributed to the change in graphene resistance; (**d**) the magnitude of V_Pulse_ for V_bias_ = 10 mV and 0 mV and the signal from the graphene for increasing gate voltage, again showing the saturation described previously; and (**e**) the magnitude of V_Pulse_ for different energies deposited in the absorber by attenuating the incident laser signal to indicate our detector’s energy sensitivity, for V_bias_ = 10 mV and 0 mV and identifying the signal attributed to the graphene.

**Figure 14:**
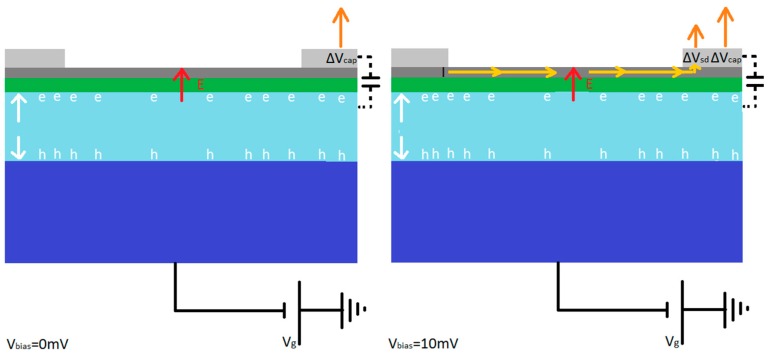
showing the different contributions to the signal that we obtain, with only a contribution from the capacitative coupling between the absorber and contact for V_bias_ = 0 mV, and the capacitative coupling contribution and from the change in voltage across the graphene providing an additional voltage pulse when V_bias_ = 10 mV.

We attribute this saturation to the generation of a depletion region in the silicon by the application of a negative gate voltage, extracting the majority carriers, holes, from the silicon gate with electrons travelling towards the insulating dielectric and the graphene. Photons are absorbed in this depletion region and generate an electron hole pair which creates a dipole aligned with the field across the silicon [93,94], whose generation controls the rise time in the signal. The charge carriers generated by the absorption of the photon scatter through the silicon in a region limited by the size of the depletion region. When this becomes large for increasingly negative gate voltages the limiting factor becomes the Shockley-Hall-Read (SRH) recombination time [95]. The V_Pulse_ peak occurs before the charge carriers recombine, with the recombination time driving the fall time of the signal. The calibrated pulsed laser was attenuated using a set of ND filters to simulate a range of X-ray energies. As expected, with increased attenuation we measured a smaller V_Pulse_, as shown in Figure 13a. The equivalent energy absorbed by the silicon absorber was then calculated, indicating the sensitivity of the detector in terms of equivalent energy of X-ray photons; this is shown in Figure 13b indicating a sensitivity equivalent to ~100 keV. When we applied the graphene drain-source current we observed a change in V_pulse_ that suggests there are two contributions to the signal. Figure 13c shows the two detector output pulses, one with, and one without the drain-source voltage (for V_bias_ = 10 mV and 0 mV respectively). The graphene peak due to the change in resistance is given by the difference between the two peaks. The invariant, larger component of the signal results from charge carriers that accumulate at the dielectric interface and are capacitively coupled to the contact. Figure 13d,e show the varying contribution that the graphene resistance change makes to the total signal with, and without a source-drain current. A schematic for the two contributions to the signal is presented in Figure 14.

Our results thus far suggest that our current devices have the potential for single photon counting at X-ray energies above 100 keV. Further work is required to improve the signal to noise ratio to improve the sensitivity to lower energies. Our detector shows a significant contribution from capacitively coupled charge carriers in the silicon, and the next step is a redesign to enhance the contribution from the change in the resistance of the graphene which, at the moment, only contributes ~10%. We are currently looking to improve this contribution by increasing the charge carrier dipole field at the graphene using a thinner insulator dielectric, or an intrinsic absorber not requiring a separate insulator, and/or by encapsulating the device to give more reliable and enhanced electrical characteristics. For instance, with an increase in the carrier mobility of the graphene, and the Dirac point located such that we obtained the maximum mobility and operated at gate voltages where we have previously observed V_Pulse_ saturation, we would operate the device where the current-gate voltage curve has a larger gradient and therefore we would expect a proportional increase in the contribution of the signal attributed to the graphene to the overall signal. In addition, including a top gate would enable variation in the drain-source current and enable the depletion region in the silicon to be created to maximise V_Pulse_.

## 7. Device Fabrication, Challenges and Progress

**Figure 15:**
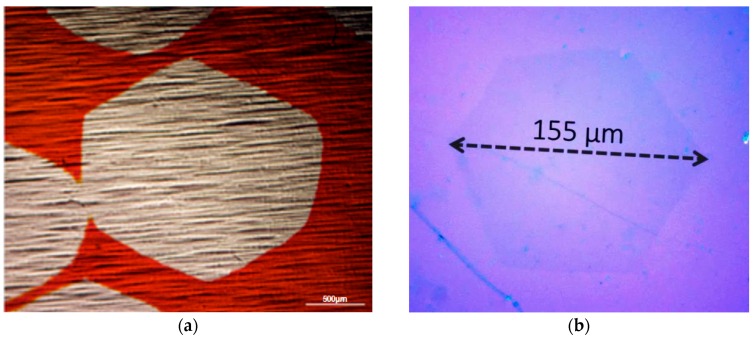
Optical micrograph of (**a**) an individual domain of mm sized CVD grown single layer graphene on Cu and (**b**) bilayer graphene on SiO_2_/Si substrates.

The devices discussed in the previous sections have specific requirements that create challenges in the fabrication process, such as graphene coverage, stacking and homogeneity. Micromechanical exfoliation provides easy access to graphene flakes and has been widely used experimentally to explore the properties for single-crystalline graphene and related device structures on the nano- to micrometre scale [96]. The main technical barrier to commercialisation is the development of manufacturing and processing techniques that fulfill the industrial demands for quality, quantity, reliability, and low cost [96,97]. A plethora of diverse fabrication methods have emerged to produce different types of graphene material. For the discussed photodetector applications the requirement is for “electronic-grade” material, in particular continuous films with detailed structural control that support high mobilities. The two main routes for manufacturing “electronic-grade” graphene films are epitaxial growth on silicon carbide (SiC) and chemical vapour deposition (CVD) [97,98]. The former is based on thermal decomposition of high-quality SiC wafers at high temperature (>1300 C) in a controlled atmosphere to control the Si sublimation [98]. As grown graphene-SiC interfaces can be modified by passivation and intercalation [99,100], which allows detailed interfacial tuning. However, the SiC route is severly limited by cost and allows no flexibility in substrate (limited to max 4” high quality SiC). Hence CVD has emerged as main industrial technique to scalably and economically synthesise high-quality graphene films [96,97]. The CVD process typically utilizes a planar catalyst film/foil, on which upon exposure to a gaseous carbon precursor at elevated temperatures a graphitic layer forms. Most widely used are transistion metal catalysts, such as Cu, Ni, Co and Pt, but also semiconductor surfaces such as Ge [101,102,103]. An increasingly detailed understanding of the CVD growth mechanisms [96,101,104,105] allows increasingly better structural control of the film microstructure, with single-crystal domains of cm dimensions already being achieved (Figure 15, [106]) and also CVD-grown AB-stacked graphene bi-layer films in the order of half-millimetre size recently reported on Cu via oxygen activation [107]. Figure 15b shows a >100 µm grain of bilayer graphene on device fabrication substrate that is readily achievable by CVD.

CVD is rapidly emerging also as industrially preferred technique for other 2D materials, such as h-BN and TMDs [108,109,110,111,112,113]. Particularly, CVD not only allows the growth of individual 2D layers but potentially also the direct growth of 2D heterostructures [114].

**Figure 16:**
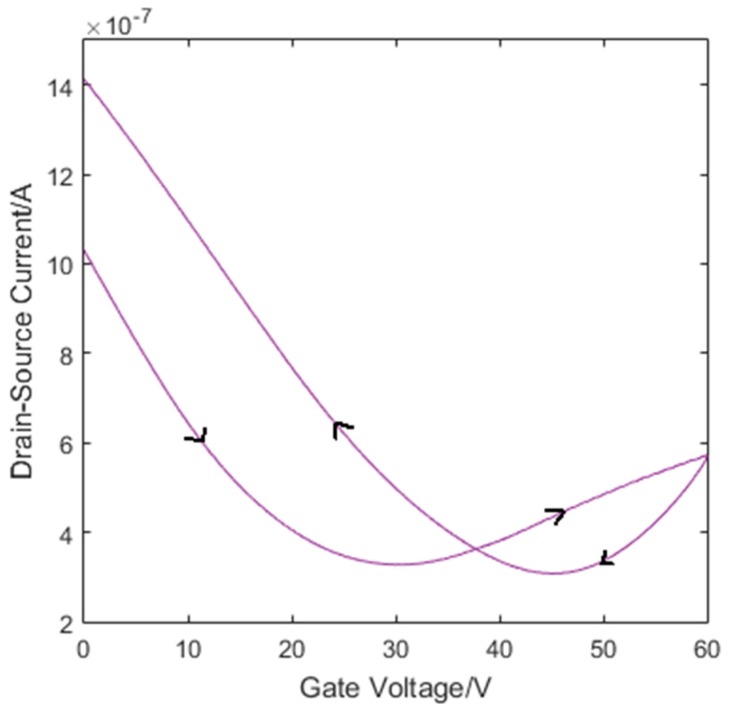
The transfer characteristics of a GFET operated in air. The large hysteresis at two Dirac points is due to trapping of charge carriers. The arrows denote the sweep direction.

Using a high quality bilayer graphene sample it is practical now to obtain mobilities of 60,000 cm^2^ *·* V^−1^ *·* s^−1^ at 1.7 K when encapsulated between h-BN [107]. When processing individual as-grown graphene layers, however, the adsorption of contaminants remains a critical issue. The operation of a GFET in air, Figure 16, results in trap states forming at the surface of graphene and at the graphene/SiO_2_ interface from moisture and OH^−^ states respectively. These trap states cause charge carriers to be trapped in these states resulting in two different Dirac points. This detriments the reliablility and instability of devices during measurements.

**Figure 17:**
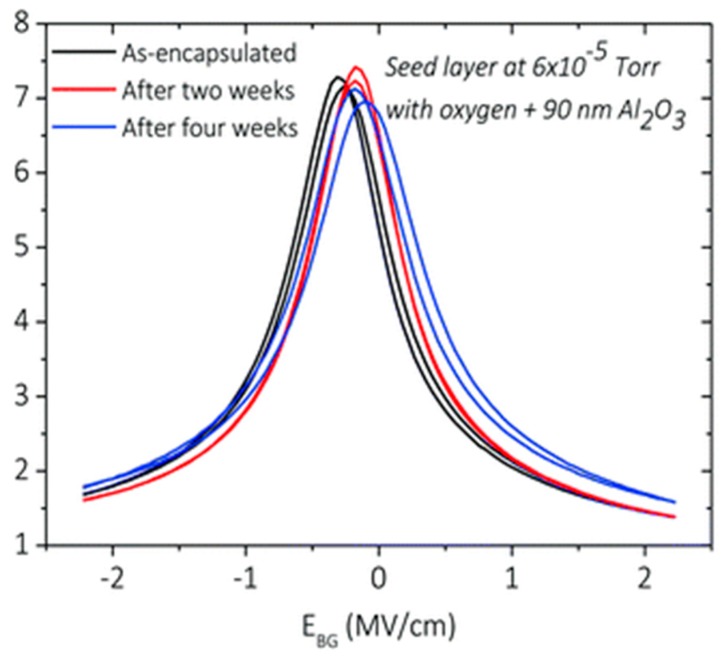
Endurance of electrical properties of encapsulated GFET measured over several weeks in ambient conditions (reproduced from [61] with permission of the Royal Society of Chemistry). The reproducibility in the characteristics is very important in photon counting applications.

To overcome such issues en route to scalable future technology it is desirable to encapsulate the device; hBN is the most promising 2D insulator for this purpose and has showed promising results in proof-of-concept devices [115,116,117]. Realising its suitability for large area CVD growth is a challenging path and hence difficult to implement in current technology. Another technique that can be used is atomic layer deposition (ALD), an industrially viable large area process that has shown complete passivation and encapsulation of large area graphene devices [61]. Recently Sagade et al. [36] has demonstrated viability of 90 nm alumina layer grown by ALD on GFET that demonstrated highly consistant device operation, Figure 17.

## 8. Conclusions

In this review we have considered the various challenges facing graphene-based single photon counting photodetectors and their prospects at a technological level. The future applications of single photon counting photodetectors requires high detection efficiency with wavelength specificity, good temporal resolution and low dark counts. Graphene’s high mobility, tunable band gap (in bilayer graphene), strong dependence of conductivity on electric field, and other properties make it particularly suitable for this application. Here graphene acts as an (indirect) photoconductor with a high gain of transconductance due to the sharp field effect in graphene. Compared with more conventional detector architectures based on charge sensing, the effective decoupling of the detector (graphene) and the absorber (substrate/electrode for X-ray/THz regime respectively) could offer potential benefits. This will also allow more flexibility in the choice of the absorber material. Graphene, therefore, provides an interesting solution for single photon counting applications due to its unique properties, which will make it more favorable than other 2D materials such as metal chalcogenides. The other advantages which graphene can provide are the ultralow noise and high speed of operation.

At visible wavelengths, current detector technologies are able to count single photons such as MKIDs and STJs, but are limited by a temporal resolution of ~1 µs. By contrast graphene photodetectors have shown detection on a femtosecond timescale. In addition, MKIDs are required to be operated at very low temperatures requiring expensive cryogenic techniques. Our simulations of bilayer graphene devices demonstrate wavelength specificity for a photon counter that can be operated over a wide range of temperatures; which can reduce the cost as well as size of an operating system, two factors crucial for implementation in space science. This may also enable more sensitive detectors, owing to the avoidance of wavelength dispersive elements, with potential applications in single photon fluorescence spectroscopy and the ability to sense multiple fluorophores simultaneously.

In this review we have also discussed future graphene-based THz detectors that have applications in areas such as security, astronomy and medical sciences. The lack of sensitive commercial devices currently limit opportunities for detection at 1.2 THz, a regime where significant scientific research could be enabled by graphene THz detectors. We have shown simulations and designs of our proposed detector that exploits the Dyakanov-Shur principle and have identified various antennae designs optimized to these frequencies. We have also discussed critical properties of graphene which may provide a future solution required for PCTI.

A number of options are available for detection of single X-ray photons, such as STJs and microchannel plate photomultipler tubes. STJs have good energy resolution, but must be operated at cryogenic temperatures, whilst MCP-PMTs have a timing resolution on the order of picoseconds, but provide very poor to no energy resolution. For graphene X-ray detectors, our experimental research with pulsed optical lasers, which simulate X-ray absorption, suggest a potential energy sensitivity of the detector equivalent to ~100 keV X-ray photons. We have also discussed the ample scope for the improvement in the design and operation of the detector to improve future sensitivity.

Effective integration of graphene at industrial scale in these different types of photodetectors critically depends on the development of integrated manufacturing pathways, in particular progress in CVD graphene (single- and bi-layer) growth technologies in terms of control over homogeneity of layers, defect density, doping and transfer to device relevant substrates. We have also highlighted the importance of interfacial control and graphene encapsulation to ensure reproducible and reliable device characteristics. Graphene photosensors have the unique capability to cover an energy range from THz to X-rays. Our simulations and experimental results, combined with continuing advances in growth and fabrication techniques suggest that graphene-based new photodetector technologies have a highly promising future.

## Figures and Tables

**Table 1:** Brief summary of a selection of photodetector technologies, with up and coming graphene-based technologies highlighted in grey followed by other potential solutions.

Detector Type [34]	Operating Temperature	Operational Wavelength	Timing Resolution	Energy Resolution $\frac{E}{\delta E}$	Responsivity	Size of Active Area	Photon Intensity
Superconducting Tunnelling Junction [23,35]	<1 K	1 nm–100 µm	1 µs	<20 (for E = 1.8 eV)	>~100 AW^−1^	~1 mm^2^	Single photon
				<6 (for E = 3.1 eV)			
				~200 (for E = 0.4 keV)			
				~500 (for E = 5.9 keV)			
Microwave Kinetic Inductance Detector [22,23,24,25,36]	0.1 K–1 K	Sub-mm and mm	~1 µs	>20	10^−7^ rad per quasi-particle	>1000 pixel array.	Single photon
Avalanche Photodiodes [37,38,39]	−20 °C	~<1 µm	40 ps+	~16 (for E = 5.9 keV)	~50 AW^−1^	<~25 mm^2^	Single photon
	−90 °C			~45 (for E = 5.9 keV)			
Transition Edge Sensors [40,41,42]	0.1 K	~1 nm	0.5 ms	~70 (for E = 0.1 keV)	~100,000 AW^−1^ on transition region	~5 cm^2^	Single photon
				~7000 (for E = 10 keV)			
Microchannel plate photomultiplier tube [33]	300 K	X-ray to IR	25 + ps	None across most of the spectrum, very poor at soft X-ray.	5–1000 mAW^−1^	>1000 mm^2^	Single photon
Ultrafast Graphene-based Photodetector. Photothermoelectric effect [18]	40–300 K	500–1500 nm	~50 fs	Photovoltage greater for lower temperatures.	~100 µAW^−1^	~10 µm	50 µW
X-ray GFET on SiC substrate. Field effect. [43,44,45,46,47,48,49]	300 K	~0.01–0.03 nm	-	10,000 (for E = 15 keV)	0.1 AW^−1^	20 µm × 4 µm	15 kV, 15 µA → 40 kV, 80 µA
X-ray GFET on Si substrate. Field effect. [43,44,45,46,47,48]	4.3 K	~0.01–0.03 nm	-	-	-	~10 µm	
Ultrafast GFET [50]. Photovoltaic effect.	300 K	1.55 µm	~25 ps (2 ps theory)	-	0.5 mAW^−1^	1 µm × 2.5 µm	3 mW
THz GFET. Dyakanov-Shur effect [51,52]	300 K	100 µm	~1 s	-	100 mVW^−1^	10 µm	-
Quantum Dot (Field Effect Transistor) [8,53]	4 K	805 nm	1 µs–1 ms	-	650 AW^−1^	15 µm	~3.5 mW
Black Phosphorus FET [54,55]	323–383 K	<940 nm	~1 ms	-	4.8 mAW^−1^	~10 µm	~500 µW
